# Light-Induced Orthogonal Fragmentation of Crosslinked Peptides

**DOI:** 10.1021/jacsau.3c00199

**Published:** 2023-08-17

**Authors:** Lars Kolbowski, Adam Belsom, Ana M. Pérez-López, Tony Ly, Juri Rappsilber

**Affiliations:** † Chair of Bioanalytics, Technische Universität Berlin, 10623 Berlin, Germany; ‡ Wellcome Centre for Cell Biology, University of Edinburgh, Edinburgh EH9 3BF, U.K.; § Si-M/″Der Simulierte Mensch″, a Science Framework of Technische Universität Berlin and Charité - Universitätsmedizin Berlin, 10623 Berlin, Germany

**Keywords:** crosslinking mass spectrometry, ultraviolet photodissociation, peptide fragmentation, MS3-triggering, orthogonal cleavage

## Abstract

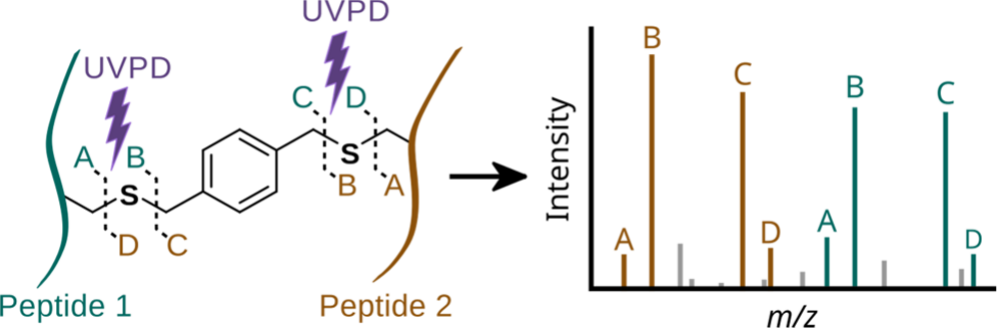

Crosslinking mass spectrometry provides pivotal information on the structure and interaction of proteins. MS-cleavable crosslinkers are regarded as a cornerstone for the analysis of complex mixtures. Yet they fragment under similar conditions as peptides, leading to mixed fragmentation spectra of the crosslinker and peptide. This hampers selecting individual peptides for their independent identification. Here, we introduce orthogonal cleavage using ultraviolet photodissociation (UVPD) to increase crosslinker over peptide fragmentation. We designed and synthesized a crosslinker that can be cleaved at 213 nm in a commercial mass spectrometer configuration. In an analysis of crosslinked *Escherichia coli* lysate, the crosslinker-to-peptide fragment intensity ratio increases from nearly 1 for a conventionally cleavable crosslinker to 5 for the UVPD-cleavable crosslinker. This largely increased the sensitivity of selecting the individual peptides for MS3, even more so with an improved doublet detection algorithm. Data are available via ProteomeXchange with identifier PXD040267.

## Introduction

Crosslinking mass spectrometry (crosslinking MS) is a potent tool to elicit molecular structures of proteins and protein complexes. It complements traditional structural biology techniques such as X-ray crystallography and cryo-EM, with distance restraints gleaned in solution from proteins and their complexes in purified systems but also in situ.^1−3^ Especially in the context of complex mixture analyses, MS-cleavable crosslinkers are preferentially used.^4^ Indeed, large-scale comparative studies between cleavable and noncleavable crosslinkers have shown that the use of cleavable crosslinkers improves the number of reliably identifiable crosslinks, especially in complex samples.^5,6^

Generally, MS-cleavable crosslinkers break apart, in multiple locations, releasing the two crosslinked peptides with attached crosslinker remnants (also called stubs). This results in a characteristic fragmentation pattern with defined mass differences between the stub fragments of each peptide. One commonly employed acquisition strategy uses these patterns to select the two peptides separately for MS3 fragmentation by searching for peaks with the specific mass difference in the MS2 (usually, the doublet of the two most common crosslinker stub fragments is used). Using this MS3-based approach, each crosslinked peptide can then be identified from a separate spectrum akin to protein identification, though at the cost of an increased acquisition time.^7^ However, recent studies have shown that the simpler and faster MS2-only approach using stepped higher-energy collisional dissociation (HCD) outperforms MS3-based approaches in the number of identified crosslinks.^6,8,9^ This can be attributed to a lack of specificity and sensitivity in selecting the peptide doublets for MS3, which currently limits MS3-based approaches.^6^

Almost all current MS-cleavable crosslinkers contain labile bonds that break during collision-induced dissociation (CID) in the mass spectrometer,^10^ effectively utilizing the same fragmentation technique for crosslinker and peptide backbone cleavage. The labile bonds in the crosslinker are intended to cleave preferentially and at lower normalized collision energies (NCE) than the peptide backbone.^11^ Yet the bond strengths of the crosslinker and peptides are too similar to ensure orthogonal cleavage of the crosslinker. This leads to harder-to-detect doublet peaks as a result of abundant peptide fragmentation. Additionally, one of the two signal doublets generated by the crosslinker cleavage can be missing, presumably as it has fragmented further.^6,12^ In fact, this double fragmentation provides additional peptide sequence coverage, which has been identified as the primary advantage of cleavable crosslinkers in MS2-based approaches.^6^ Double fragmentation, however, is detrimental to MS3-based approaches, where crosslinker cleavage is the critical MS2 process. Access to a cleaner, more orthogonal way of cleaving the crosslinker with little peptide backbone cleavage would therefore substantially advance MS3-based acquisition strategies. Indeed, a UVPD-cleavable system has been proposed for crosslinking, although it remains to be tested on proteins and is not yet implemented on a commercial mass spectrometer, due to the required 355 nm setup.^13^

In contrast to collisional-activation-based methods (CID and HCD), ultraviolet photodissociation (UVPD) achieves bond dissociation using photons.^14^ The photons are generally produced by lasers at specific wavelengths and can be applied over varying excitation times. Laser wavelength determines the chromophores targeted and is therefore critical for the desired application. 213 nm UVPD is so far mostly used in top-down proteomics and has been shown to elicit only sparse peptide backbone fragmentation in bottom-up experiments, especially at short excitation times.^15^ Also, 193 nm UVPD is used for fragmentation of peptide bonds including crosslinked peptides.^16^ The energy associated with UVPD photon absorption is rapidly redistributed through vibrational relaxation such that dissociation frequently requires multiphoton activation. In contrast, carbon–sulfur bonds of benzyl mercaptans are highly susceptible to rapid, direct bond dissociation following UVPD irradiation at 213 nm^17^ and at 266 nm.^18^ Introducing such chromophores into a crosslinker might lead to a stark preference for cleaving the crosslinker over the peptides. This would lead to cleaner spectra with easier-to-detect doublets and thus higher success in triggering the correct peaks for MS3 fragmentation.

**Figure 1:**
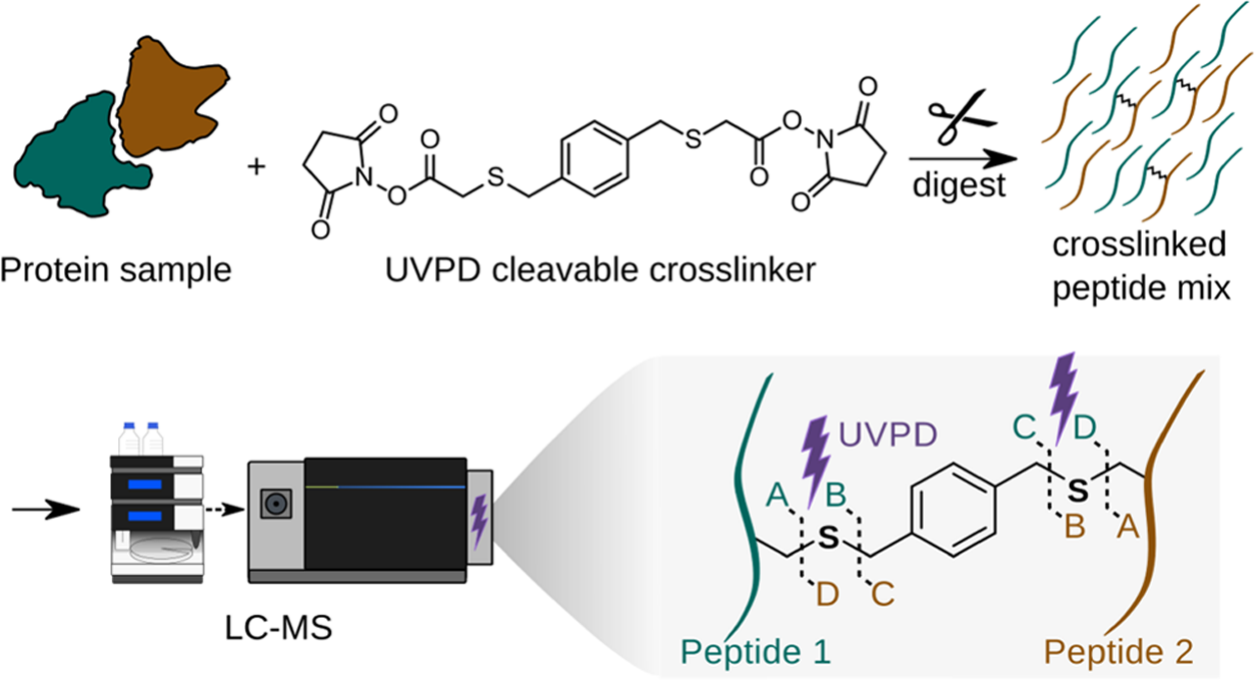
UVPD-cleavable crosslinker workflow.

We here designed and synthesized a crosslinker that might be cleaved at 213 nm UVPD (Figure 1). We compared our UVPD-cleavable crosslinker (UCCL) to the established CID-cleavable crosslinkers DSSO^19^ and DSBU^20^ with respect to the detectability of signature peptide doublets. Further, we evaluated and compared the sensitivity and specificity of MS3 precursor selection of UCCL versus DSSO. Additionally, we used the results from our analysis to design and test an improved doublet detection algorithm to further increase the specificity and sensitivity of MS3-based approaches.

## Results and Discussion

### Development of UCCL, a UVPD-Cleavable Crosslinker

C–S bond-selective photodissociation with 213 nm is enhanced when sulfur is removed from an aromatic system by one sp^3^ carbon but hindered when the sulfur is incorporated directly onto an sp^2^ carbon.^17^ This suggested to us that a benzyl mercaptan could provide a reasonably effective UVPD-cleavable specific building block that could be incorporated into a protein-reactive crosslinking reagent.

**Figure 2:**
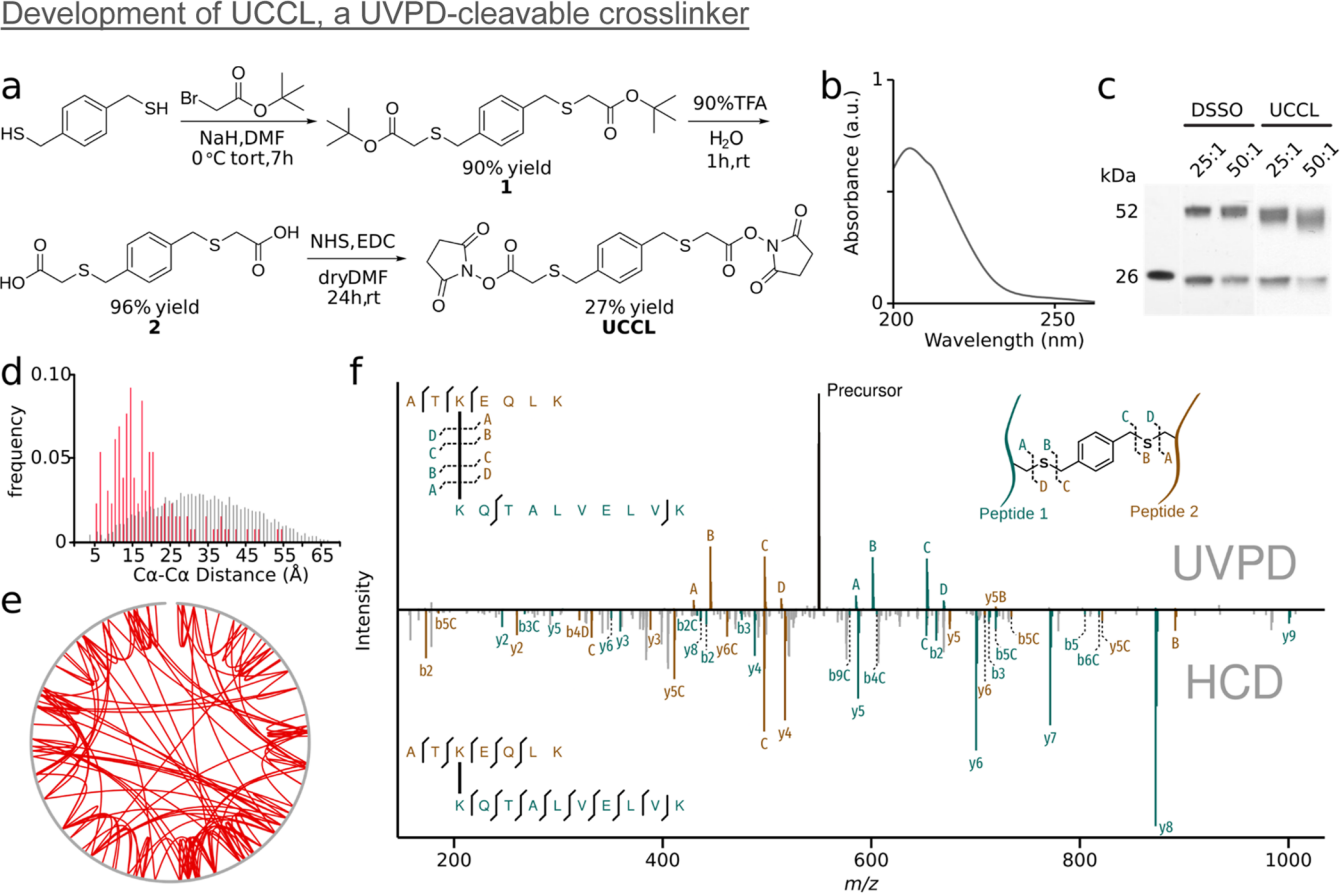
UVPD-cleavable crosslinker synthesis and crosslink feasibility. (a) Synthesis of UCCL. (b) Absorbance spectrum of UCCL in a nonpolar solvent (hexane) (c) Dimeric GST crosslinked with either DSSO or UCCL using two different molar ratios for both crosslinking reagents. (d) HSA distogram showing residue–residue Cα–Cα distances of UCCL-crosslinked residues (in red) as observed in the crystal structure PDB 1AO6, against a random Cα–Cα distance distribution (in gray). (e) HSA crosslink network (*n* = 135, 5% FDR). (f) Butterfly plot of a UVPD spectrum (top; 20 ms excitation time) and HCD spectrum (bottom; stepped HCD 26;28;30 NCE) of the same crosslinked precursor from LC-MS analysis of UCCL-crosslinked HSA. Fragment labels for peaks >1% base peak intensity are shown.

We synthesized UCCL in a very straightforward three-step synthesis using commercially available starting materials (Figure 2a). In short, 1,4-Benezenedimethanethiol was alkylated using *tert-*butyl bromoacetate, the *tert*-butyl groups were removed using TFA (in near quantitative yield), and the resulting carboxylic acids were activated to provide two protein/peptide reactive NHS ester functional groups (overall yield 23%). The resulting UVPD-cleavable crosslinking reagent, UCCL, contains a symmetric pair of two MS-labile C–S bonds flanking the aromatic chromophore. Measuring the UV absorbance of UCCL showed strong absorbance around 213 nm (Figure 2b).

UCCL has a spacer length of 13.2 Å, which makes it effective for producing highly informative distance constraints. We first demonstrated this by crosslinking the homodimeric protein, glutathione S-transferase (GST), in a crosslinker titration (Figure 2c). Homodimeric GST forms a crosslinked dimer upon crosslinking. The extent of crosslinked dimer produced corresponds to the amount of crosslinking achieved and, therefore, also gives an indication of crosslinking efficiency. Furthermore, we carried out a direct crosslinking comparison with DSSO and found that at equimolar concentrations, UCCL allows highly comparable, if not even greater, amounts of crosslinking. We next assessed the suitability of UCCL for more general crosslinking MS analysis by crosslinking human serum albumin (HSA) and subjecting crosslinked protein to our standard crosslinking MS pipeline^21^ but using only HCD-MS2 fragmentation. We identified 135 unique residue pairs at 5% link-level FDR (false-discovery rate).^22^ When fitted to the crystal structure of HSA (PDB|1AO6), the median measurable Cα–Cα distance was 16.0 Å (Figure 2d,e). Next, we applied UCCL for crosslinking *Escherichia coli* lysate. Following SEC fractionation of resulting tryptic peptides and subsequent LC-MS analysis (7 LC-MS acquisitions), we identified 1884 unique residue pairs over 521 proteins at 5% link-level FDR. Having concluded that UCCL performance in our standard crosslinking MS pipeline is highly comparable to other NHS ester-based homobifunctional crosslinker reagents, we proceeded to assess MS-cleavability of crosslinked peptide pairs.

We expect four cleavage sites in the crosslinker resulting in four peptide stub fragments (A, B, C, and D) per peptide (Figure 1). Indeed, UCCL-crosslinked peptides subjected to UVPD show distinct and prominent crosslinker cleavage ions with little peptide backbone cleavage (Figure 2f, top). In contrast, HCD leads to standard peptide backbone fragmentation with scarce cleavage of the C–S bond (Figure 2f, bottom). To find a suitable laser excitation time that maximizes stub fragments, we acquired UCCL-crosslinked HSA as a model protein. We screened a wide range of excitation times (1–200 ms UVPD) using a dual MS2 acquisition strategy that utilizes additional HCD spectra for identification.^15^ We found the ratio of detectable stub fragment doublets in our crosslink spectrum matches (CSMs) starting to plateau at 20 ms and dropping off again after 50 ms of excitation time (Figure S1a). Note that the precursor cleavage efficiency increases with increased excitation time, from 41% (20 ms) to 71% (50 ms) (Figure S1b). The B–C doublet could be detected most frequently, with 95% of CSMs containing at least one peptide doublet and 87% containing both peptide doublets at 20 ms. We noticed that A, B, C, and D ions were observed with UVPD-typical variants arising from the presence or absence of extra hydrogen.^23^ This results in multiple possible delta masses between stub fragments that we consider during acquisition and data analysis (Figure S2a,c).

### Mass Spectrometric Fragmentation of UCCL-Linked Peptides

**Figure 3:**
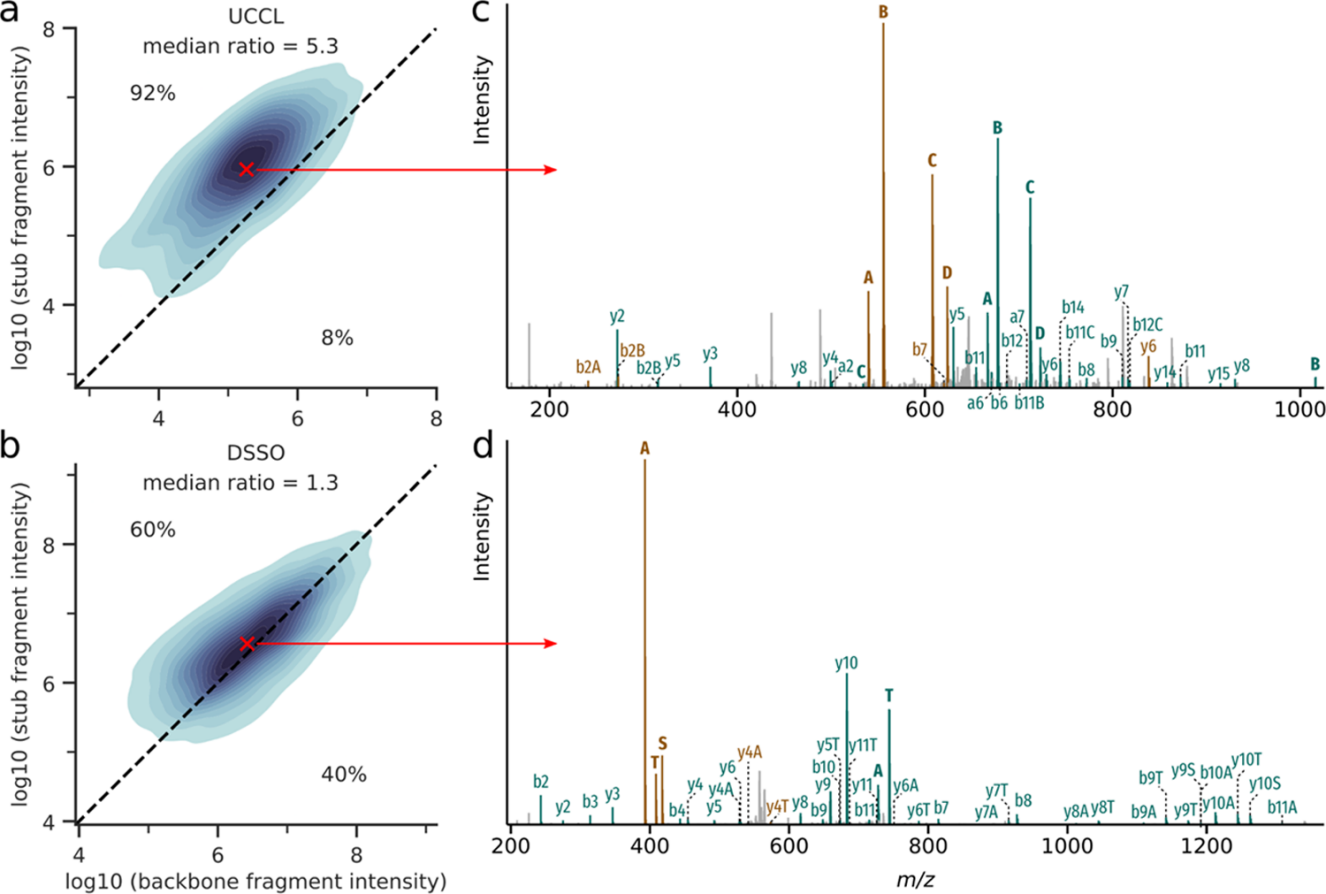
Orthogonal crosslinker cleavage. (a, b) Kernel density estimate distribution of the peptide backbone fragment intensity vs the cleaved crosslinker peptide stub intensity for the (a) UCCL and (b) DSSO datasets. The spectra closest to the geometric median of the distribution are marked in red. (c) Representative spectrum (closest to the geometric median in panel a) of a UCCL crosslinked peptide (AKLHDYYK-KLMTEFNYNSVMQVPR) fragmented with UVPD (precursor peak was removed during data analysis, see Methods section). (d) Representative spectrum (closest to the geometric median in panel) (b) of a DSSO crosslinked peptide (SRIAKR-NQAEEELIKAQK) fragmented with CID.

We next expanded our observation of UCCL behavior toward our UCCL-crosslinked *E. coli* lysate as a complex biological sample. We analyzed SEC fractions using dual MS2 acquisition of 20 ms UVPD and HCD on the same precursor, with additional MS3-triggering on the 5 most common B-C variant doublet delta masses (Figure S2b). From the HCD spectra, we could identify 1283 CSMs at a 5% CSM-level FDR. We then annotated all respective UVPD spectra with the most commonly occurring peptide backbone fragments (a-, b- and y-ion series)^15^ as well as the four peptide stub fragments (A, B, C, and D). We then plotted the summed intensities of the stub versus backbone fragments (Figure 3a) to systematically assess the orthogonality of crosslinker cleavage and peptide backbone cleavage under UVPD conditions. To assess the bias of UCCL for crosslinker cleavage against that of a CID-cleavable crosslinker, we also analyzed a DSSO dataset^24^ which employed a CID-MS2-MS3-ETD-MS2 acquisition strategy.^12^ Analogous to our dataset, we focused on the low-energy CID spectra, which had been optimized for preferential crosslinker cleavage. We annotated the respective DSSO peptide stub fragments (A, S, and T) and the most common CID peptide backbone fragments (b- and y-ion series). UCCL outperformed DSSO in peptide stub fragments. In 92% of CSMs from the UCCL dataset, the peptide stub fragments have a higher summed intensity than the backbone fragments, compared to only 60% of CSMs in the DSSO dataset. The median ratio of the summed stub to backbone fragment intensities is 4 times higher for UCCL than for DSSO (5.3 and 1.3, respectively). Overall intensities for UCCL are lower due to the relatively high amount of unfragmented precursors. This could be increased by using longer excitation times, increased laser power, or future design of better chromophores. To extract representative spectra for each dataset, we calculated the geometrical median for both distributions and selected the closest data point as representative of each dataset (Figure 3c,d). Beyond the statistical analysis above, viewing these two representative spectra side by side shows clearly that the peptide stub fragments from UCCL are more prominent than their DSSO counterparts.

**Figure 4:**
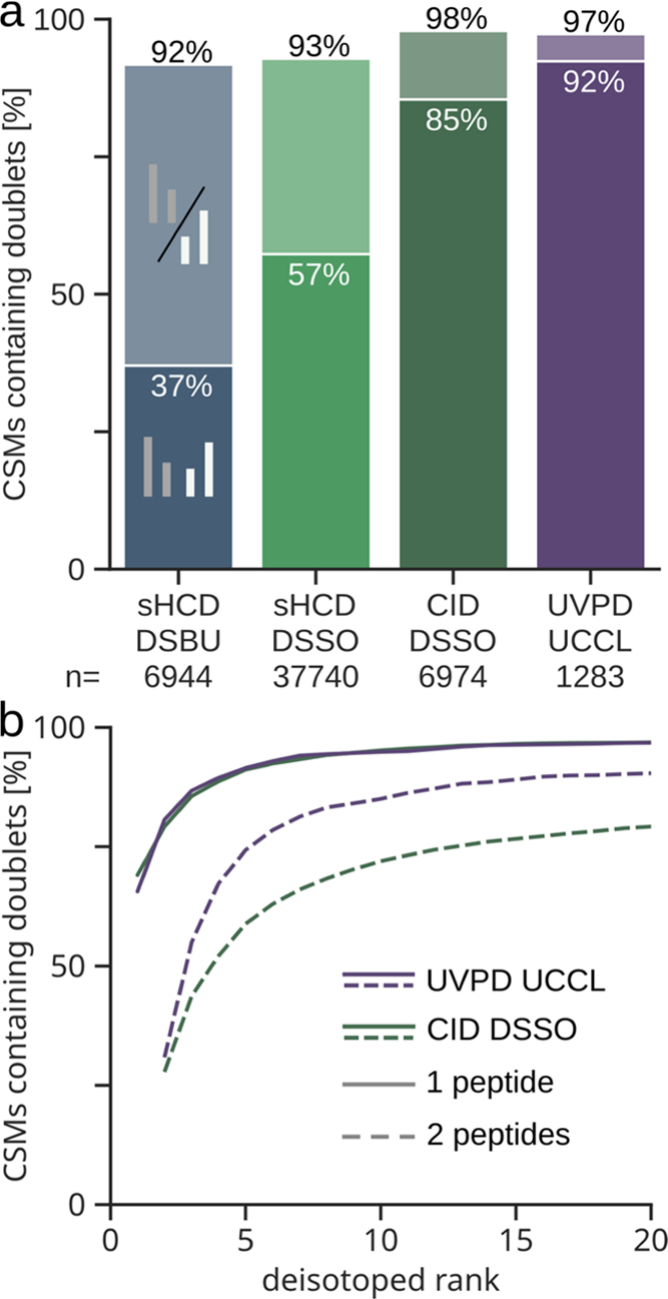
Prevalence and prominence of doublets in CSMs. (a) Proportion of identified CSMs that contain one (lighter shade colors) or both (darker shade colors) peptide doublets in each dataset (5% CSM-level FDR). (b) Proportion of CSMs containing doublets passing different intensity rank cutoffs for the UVPD-UCCL and CID-DSSO datasets.

Next, we evaluated the prevalence and prominence of B-C doublets systematically for all CSMs in our UCCL dataset and compared them to the results of DSSO and DSBU with CID-MS2-MS3 as well as stepped HCD-MS2 acquisition methods.^6^ Doublets for one peptide could be detected reliably across CID-cleavable crosslinker datasets independent of the fragmentation method (Figure 4a). As one might expect, detection of DSSO doublets for both crosslinked peptides is higher in acquisition schemes utilizing a low-energy NCE CID scan, which has been designed for preferential crosslinker cleavage. Similar to the CID-DSSO results, we found a very high proportion of CSMs containing at least one peptide doublet in our UCCL data (97%). In detecting both peptide doublets, the results of the UCCL dataset surpass the best DSSO dataset (92 vs 85% of CSMs contained both doublets). Detection of both peptide doublets is especially important for MS3-based acquisition approaches because, without a detectable doublet, no MS3 can be triggered, meaning no separate fragmentation sequence information can be obtained for this peptide.

Aside from detectability, peptide doublet intensity is also of interest. The higher the intensity of the peaks of interest compared to other peaks in the spectrum, the easier it becomes to pick out the correct peaks for MS3 fragmentation. To compare this, we applied several intensity-based rank cutoffs to the spectra and checked what proportion of our CSMs still contained the peptide doublets (Figure 4b). For the more intense doublet, both UCCL and DSSO show very similar curves, with 66 and 69% of CSMs, respectively, in which the doublet contains the most intense peak of the spectrum. Almost all CSMs contain this doublet among the top 20 peaks (both 97%). Differences between UCCL and DSSO become visible when comparing how many CSMs contain both peptide doublets at high-intensity ranks in the spectrum. 74% of the UCCL CSMs contain both peptide doublets among the top 5 ranks, whereas in the DSSO dataset, this is only the case for 59% of CSMs. Including the top 20 ranks, almost all CSMs of the UCCL dataset contain both peptide doublets (90%), whereas this only applies to 79% of CSMs from the DSSO dataset. In conclusion, UCCL performs comparably to DSSO concerning the visibility of the higher intense doublet but outperforms DSSO with regard to the visibility of the second peptide doublet. So, not only the detectability but also the intensity rank of UCCL peptide stub fragments is better than that of DSSO or DSBU (Figures 4 and S3), suggesting UCCL as superior in MS3-triggering to gain sequence information for both crosslinked peptides. To maximize information gain from MS3 would require optimizing the acquisition parameters in this respect, which was not done here. However, we investigated if UVPD leading to radical formation in MS2 affected the type of observed sequence ions in MS3. Comparing the frequency of c-, x- and z-ions between our *E. coli* UCCL data and the synaptosome DSSO dataset did not lead to obvious differences (Figure S5). This suggests that UVPD in MS2 does not influence the fragmentation behavior of the peptides in MS3.

### Advancing MS3-Based Crosslinking MS Strategies

**Figure 5:**
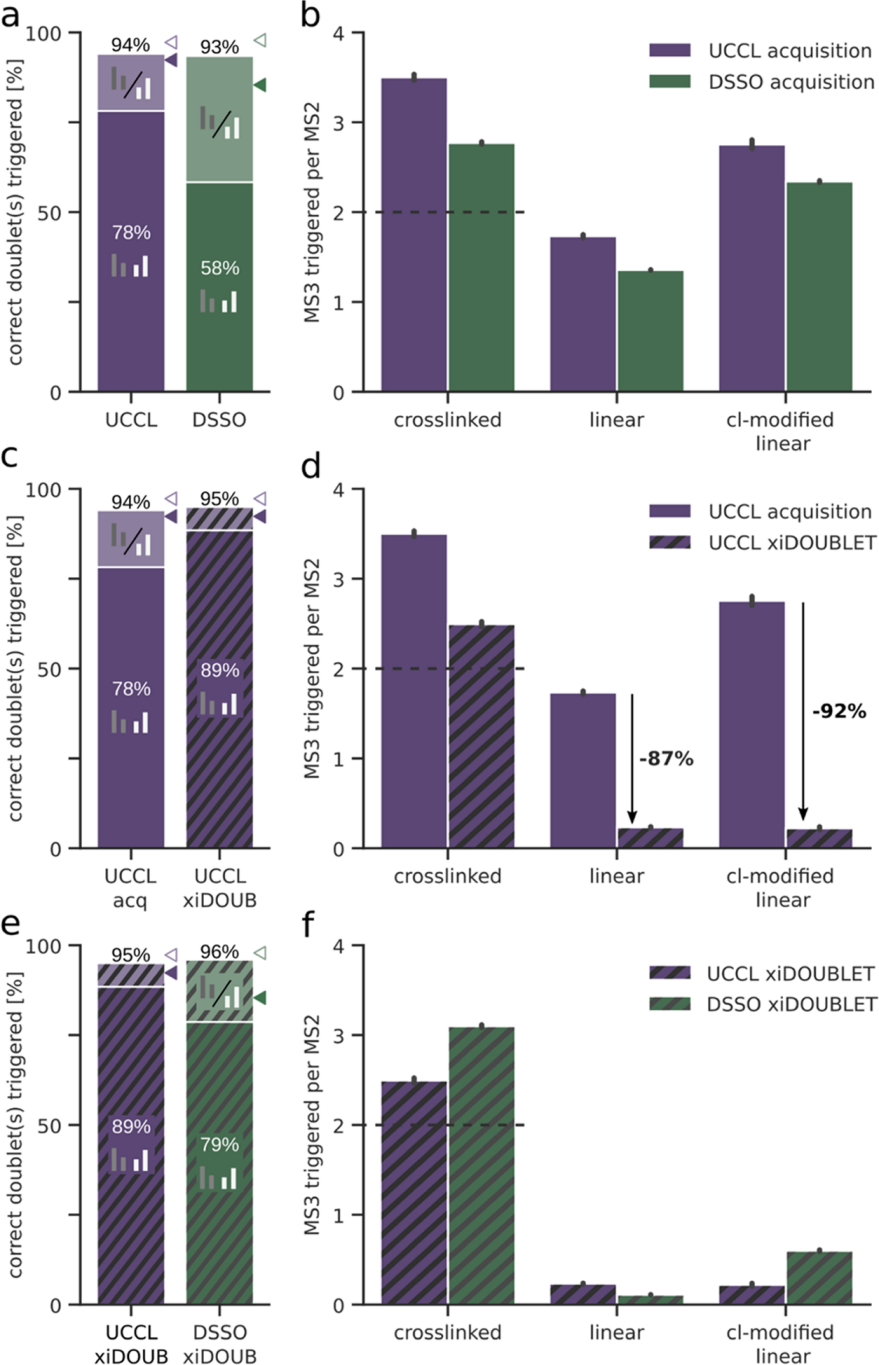
Sensitivity and specificity of MS3-triggering. (a, c, e) Proportion of CSMs passing 5% unique CSM-level FDR having at least one (lighter shade colors) or both (darker shade colors) peptide doublets correctly triggered for MS3 in the respective datasets. Triangles show the percentage of CSMs in which at least one (lighter shade colors) or both (darker shade colors) peptide doublets could be annotated. (b, d, f) Number of triggered MS3 scans per MS2 scan for CSMs, linear peptide spectrum matches, and crosslinker (cl) modified linear peptide spectrum matches, respectively (nonunique PSMs/CSMs passing 5% CSM-level FDR). Error bars show the 0.95 confidence interval.

CID-cleavable crosslinkers suffer from a lack of sensitivity and specificity in triggering the correct peaks for MS3, hampering MS3-based acquisition strategies.^6^ To evaluate the relative sensitivity of MS3-triggering when using DSSO or UCCL, we checked how often an MS3 precursor matched a peptide stub fragment in the respective MS2 spectra. Even though both peptide doublets could be annotated in 85% of the CID-DSSO CSMs (Figure 4a), only 58% had MS3 spectra acquired for both crosslinked peptides (Figure 5a). For UCCL, 78% of CSMs had MS3 spectra acquired for both crosslinked peptides, demonstrating the advantage of the orthogonal fragmentation approach.

The additional acquisition time cost due to MS3 spectra acquisition is one of the downsides of MS3-based approaches.^6,7^ This can lead to a lower sampling rate of precursors from the MS1, which in turn negatively affects the number of crosslink identifications. It is desirable therefore to acquire only as many MS3 spectra as necessary, i.e., acquisition of ideally only two MS3 spectra per crosslinked precursor (one for each peptide) and none for linear peptides. We evaluated the specificity of MS3-triggering for all (nonunique) CSMs, linear PSMs, and crosslinker-modified linear PSMs for the UCCL and DSSO datasets (Figure 5b). Note that crosslinker modification leads to a stub doublet. Both datasets show relatively poor specificity in MS3-triggering with a high average number of MS3 spectra acquired for linear PSMs (DSSO: 1.36 and UCCL: 1.73) and an even higher number for crosslinker-modified linear PSMs (DSSO: 2.34 and UCCL: 2.75). Surprisingly, the cleaner fragmentation spectra in the UCCL acquisition performed even a bit worse than the DSSO dataset.

This might be attributed to the choice of nonideal MS3-triggering parameters. Consequently, using an advanced doublet detection algorithm could help to alleviate the specificity problem of MS3-based acquisition strategies. We employed our novel xiDOUBLET algorithm^25^ with an additional filter for multiple doublet triggers per ±1.5 *m*/*z* window to prevent multiple MS3 triggers on the same peptide doublet. This additional filter is specifically relevant for UCCL because we searched for several doublet delta masses due to the hydrogen shifts observed. In our acquisition, we used the exclusion list feature on the MS3 precursor selection from the vendor software, which should achieve a similar result. Apart from this new feature, we varied two main parameters that can also be set in the vendor software, namely, the mass tolerance for matching the doublet delta mass and the number of delta masses considered.

Optimization of doublet match mass tolerance and number of delta masses considered resulted, for our dataset, in a 5 ppm mass tolerance and using only the three most commonly observed delta masses (mass shifts: 0, +1H, and +2H, Figure S4). Using these optimized parameters, our doublet selection algorithm vastly improved the specificity in the UCCL dataset, reducing the average number of MS3 spectra triggered for (linear) PSMs to 0.23 and crosslinker-modified PSMs to only 0.22, thus reducing the unwanted triggering of MS3 by 87 and 92%, respectively (Figure 5d). Additionally, the average number of MS3 spectra triggered for CSMs could be brought closer to the ideal value of 2, saving additional acquisition time. Using our doublet detection algorithm had the additional benefit of increasing the proportion of CSMs that had both doublets correctly triggered from 78.2 to 88.5%. This almost completely closes the gap to the theoretical maximum of 92% of CSMs with annotated doublets (Figure 4a).

A direct comparison of the xiDOUBLET results of the two datasets shows that while results for both datasets move toward their respective theoretical maximum of CSMs that contained annotatable doublets, UCCL still outperforms DSSO with 89% of CSMs for which both peptides were triggered correctly against only 79% in the DSSO data (Figure 5e). In terms of specificity, both datasets perform similarly well using the xiDOUBLET algorithm (Figure 5f). In the UCCL data, fewer unnecessary MS3s were triggered in the crosslinked and crosslinker-modified category, whereas in the DSSO dataset, slightly fewer MS3 spectra were triggered in the linear category.

A limitation of our approach is the acquisition speed that can be achieved when using UVPD on current commercial mass spectrometers. While CID-based approaches can be used with parallel acquisition, this is not implemented for UVPD yet. Here, the vendor (Thermo Fisher) is required to take action so that UVPD can be applied in routine crosslinking studies and, with this, help progress biological studies. Our work using a commercial instrument has the advantage of easier take-up across laboratories but comes at the expense of depending on the vendor for necessary adaptations. Demonstrating the proof of principle of UVPD in crosslinking MS opens the field to further technical developments to ultimately reach biology.

## Conclusions

Using 213 nm UVPD in combination with a UV-absorbing chromophore integrated into a crosslinker introduces orthogonal cleavage of the crosslinker and peptide backbone. This alleviates major caveats of conventional CID-cleavable crosslinkers. Orthogonal cleavage improved sensitivity in the selection of the peptide stub fragments for MS3 fragmentation. The possible specificity gain is then fully exploited by our improved doublet selection algorithm. Orthogonal cleavage is a general concept that may improve not only the analysis of crosslinked peptides but also that of peptides linked to other biomolecules, including DNA, RNA, or saccharides.

## Methods

All materials and methods used, including crosslinker reagent synthesis, sample crosslinking and preparation for MS analysis, MS acquisition strategies, and data analysis, are described in full detail in the Supporting Information.

### *E. coli* Lysate Crosslinking and LC-MS Analysis

Prepared *E. coli* lysate was crosslinked using 0.85 mM UCCL, incubating at room temperature for 45 min. Crosslinked proteins were precipitated in ice-cold acetone and digested using trypsin. Crosslinked peptides were enriched by size-exclusion chromatography using a Superdex Peptide 3.2/300 column (GE Healthcare) and subsequently analyzed (see pages S4–S6) using an Ultimate 3000 RSLC nano system (Dionex, Thermo Fisher Scientific, Germany) coupled online to an Orbitrap Fusion Lumos Tribrid mass spectrometer equipped with an EasySpray source and a UVPD module (Thermo Fisher Scientific, Germany) featuring a 213 nm solid-state Nd:YAG laser head (CryLaS GmbH).

### Data Analysis

Mass spectrometry raw data were preprocessed using a custom Python script (https://github.com/Rappsilber-Laboratory/preprocessing), converting files to the MGF file format using MSconvert^26^ with subsequent *m*/*z* recalibration of both MS2 precursor and fragment peaks by employing a linear peptide search to determine the median mass error. The spectra from acquisitions containing multiple MS2 or MS3 were split into separate MGF files for each fragmentation method and MS level. The recalibrated HCD-MS2 spectra were then searched using xiSEARCH^21^ 1.7.6.1 against UniProt protein sequences (see page S7). A 5% CSM-level FDR (minimum peptide length of 5 amino acids) was applied. UVPD spectra were then annotated using pyXiAnnotator (see page S8).

## Supporting Information Available

Methods regarding sample preparation, LC MSn analysis, and data analysis. Additional figures about UCCL stub fragment analysis; observed hydrogen shift variants of peptide stub fragments; version of Figure 4b including also the sHCD datasets; results of xiDOUBLET parameter optimization; ^13^C NMR and ^1^H NMR spectra for all compounds. (PDF) All proteomics data have been deposited to the ProteomeXchange Consortium via the PRIDE^27^ partner repository with the dataset identifiers PXD040267 and 10.6019/PXD040267 (PDF)
