# Supplementary material for: Light-Induced Orthogonal Fragmentation of Crosslinked Peptides
Source: JACS Au. 2023 Aug 17;3(8):2123–30. doi: 10.1021/jacsau.3c00199 (PMC10466327; doi:10.1021/jacsau.3c00199)
Supplement: Supplementary file 1 — au3c00199_si_001.pdf [file au3c00199_si_001.pdf]

Supporting Information for

Light-Induced Orthogonal Fragmentation of Crosslinked Peptides

Lars Kolbowski[1,$], Adam Belsom[1,$], Ana M. Pérez-López[1], Tony Ly[2,#], Juri Rappsilber[1,2,3,*]

[1] Technische Universität Berlin, Chair of Bioanalytics, 10623 Berlin, Germany
[2] Wellcome Centre for Cell Biology, University of Edinburgh, Edinburgh EH9 3BF, UK
[3] Si-M/"Der Simulierte Mensch", a Science Framework of Technische Universität Berlin and Charité - Universitätsmedizin Berlin, 10623 Berlin, Germany
[#] Present address: Centre for Gene Regulation and Expression, School of Life Sciences, University of Dundee, Dundee, DD1 5EH, UK

[$] L.K. and A.B. contributed equally to this work.
* Correspondence to juri.rappsilber@tu-berlin.de

This file includes:

- Methods including chemicals and materials, sample preparation, LC MSn analysis, and data analysis
- Figures S1-11
- Supplemental references

Methods

Chemicals and Materials

Chemicals and solvents were obtained from Fisher Scientific, Sigma-Aldrich or VWR International Ltd. NMR spectra were recorded at ambient temperature on a 500 MHz Bruker Avance III spectrometer. Chemical shifts are reported in parts per million (ppm) relative to the solvent peak. High-Resolution Mass Spectrometry was measured in a Bruker MicroTOF II. R_f values were determined on Merck TLC Silica gel 60 F254 plates under a 254 nm UV source. Purification of compounds was carried out by flash column chromatography using commercially available silica gel (220-440 mesh, Sigma-Aldrich) or Chromabond C18 SPE Cartridges (6 mL/1000 mg, Fisher Scientific). LC-MS was performed using an Agilent 1200 system with a Bruker MicroTOF II. Method: eluent A: water and formic acid (0.1 %); eluent B: acetonitrile and formic acid (0.1 %); A/B = 95:5 isocratic 0.5 min, 95:5 to 0:100 in 4.5 min, isocratic 2 min, 0:100 to 95:5 in 0.5 min, and isocratic 2.5 min (flow = 0.2 mL/min). The purity of compounds was >95% as measured by HPLC, NMR and TLC. No unexpected or unusually high safety hazards were encountered.

Synthesis of Di(*tert*-butyl)-2,2'-[1,4-phenylenebis(methylenethio)]bis-acetate (1)

1,4-Benzenedimethanethiol (250 mg, 1.47 mmol) was dissolved in dry DMF (10 mL) under a nitrogen atmosphere and cooled to 0 °C. NaH (60% suspension oil, 130 mg, 3.23 mmol) was then added, and the resulting solution was stirred for 30 minutes. *tert*-Butyl bromoacetate (477 µL, 3.23 mmol) was dissolved in dry DMF (0.5 mL). The solution was added dropwise and the resulting mixture stirred at room temperature for 7 h. The reaction was quenched by the slow addition of water. Solvents were then removed under reduced pressure and the crude purified via flash chromatography, eluting with AcOEt:Hexane (1:20). R_f = 0.20 (5% AcOEt in Hexane) to yield **1** as a pale oil (526 mg, 90% yield). **^{1}H NMR** (500 MHz, DMSO) δ 7.26 (s, 4H), 3.79 (s, 4H), 3.06 (s, 4H), 1.42 (s, 18H). **^{13}C NMR** (126 MHz, DMSO) δ 168.96, 136.38, 128.97, 128.95, 80.75, 35.03, 33.34, 27.60. **LC-MS** (*m/z*): $[M+Na]^+$ 421.1503. **HRMS (ESI)** *m*/*z* $[M+H]^+$ calcd for $C_{20}H_{31}O_4S_2$, 399.16583; found, 399.16617.

Synthesis of 2,2'-[1,4-phenylenebis(methylenethio)]bis-acetic acid (2)

Compound **1** (526 mg, 1.32 mmol) was dissolved in a TFA solution (90% in water) and stirred for 2 h at room temperature. The mixture was then concentrated at reduced pressure. The crude was purified using Chromabond C18 SPE Cartridges, 6 mL/1000 mg (30% MeOH in H_2O) to yield **2** as a pale oil (362 mg, 96% yield). **^{1}H NMR** (500 MHz, DMSO) δ 12.60 (s, 2H), 7.26 (s, 4H), 3.79

(s, 4H), 3.11 (s, 4H). **^{13}C NMR** (126 MHz, DMSO) δ 171.24, 136.48, 129,00, 35.08, 32.63. **LC-MS** (*m/z*): $[M + Na]^+$ 308.9943. **HRMS (ESI)** *m*/*z* $[M-H]^-$ calcd for $C_{12}H_{13}O_4S_2$, 285.02610; found, 285.02559.

Di(N-succinimidyl)-2,2'-[1,4-phenylenebis(methylenethio)]bis-acetate (3, UCCL)

Compound **2** (362 mg, 1.26 mmol) and N-hydroxysuccinimide (290 mg, 2.52 mmol) were dissolved in DMF dry (6 mL) under nitrogen atmosphere, followed by addition of 1-ethyl-3-(3-dimethylaminopropyl) carbodiimide hydrochloride (EDC·HCl) (481 mg, 2.52 mmol). The mixture was stirred for 24 h at room temperature. The solvent was evaporated *in vacuo* and the solid was dissolved in DCM (20 mL), which was then washed with sat. $NaHCO_3$ (2 x 10 mL), 10% citric acid (2 x 10 mL) and brine. The organic layer was dried ($MgSO_4$) and the solvent was evaporated *in vacuo*, yielding UCCL (**3**) as a white solid (163 mg, 27%). R_f = 0.43 (5% MeOH in CH_2Cl_2). **^{1}H NMR** (500 MHz, DMSO) δ 7.31 (s, 4H), 3.92 (s, 4H), 3.62 (s, 4H), 2.85 (s, 8H). **^{13}C NMR** (126 MHz, DMSO) δ 170.10, 166.34, 135.94, 129.24, 34.91, 29.13, 25.49, 25.21. **HRMS (ESI)** *m*/*z* $[M]^+$ calcd for $C_{20}H_{20}N_2O_8S_2$, 480.06556; found, 480.06540.

Sample Preparation

Crosslinking GST – Dimeric GST (equine liver; Sigma-Aldrich, St. Louis, MO, USA) was buffer exchanged using an Amicon spin filter (3 kDa molecular weight cut-off; Merck, Darmstadt, Germany) into crosslinking buffer (20 mM HEPES-OH, 20 mM NaCl, 5 mM $MgCl_2$), to a protein concentration of 1.5 mg/mL. Mixing ratios of UCCL to GST were titrated using crosslinker-to-protein weight-to-weight ratios of 0.06:1, 0.13:1, 0.25:1, 0.5:1, 1:1, 2:1, 4:1 and 8:1. Mixing ratios of DSSO to GST were titrated using equivalent molar ratios, equating to crosslinker-to-protein weight-to-weight ratios of 0.05:1, 0.1:1, 0.2:1, 0.38:1, 0.75:1, 1.5:1, 3:1 and 6:1. GST (2 µg) was crosslinked with 0.5 µL of UCCL (starting concentration, 63 mM) dissolved in DMF. Crosslinking was performed for 1h at room temperature, at a protein concentration of 0.2 mg/mL, in 10 µL reaction volumes. The crosslinking reaction was quenched by adding 0.5 µL sat. ABC and mixing for 15 min at room temperature. The resulting crosslinked mixture was separated on a NuPAGE 4-12% Bis-Tris gel using MES running buffer and Coomassie blue stain.

Crosslinking HSA – Lyophilized HSA was reconstituted in a crosslinking buffer (20 mM HEPES-OH, 20 mM NaCl, 5 mM $MgCl_2$, pH 7.8). Crosslinking was carried out at a protein concentration of 0.5 mg/mL (50 µg HSA in 100 µL reaction volumes) using crosslinker-to-protein w/w ratios of 3.1:1 for DSSO and 3.9:1 for UCCL. Crosslinking was carried out for 1h at room temperature and was subsequently quenched by adding 5 µL sat. ABC and incubation for 10 minutes at room

temperature. Crosslinked HSA was separated on a NuPAGE 4-12% Bis-Tris gel using MES running buffer and Coomassie blue stain. HSA monomer bands were excised from the gel, proteins reduced with 20 mM DTT, alkylated using 55 mM IAA, and digested overnight at 37 °C using trypsin. The crosslinked peptide digest was acidified to a pH of 2-3 with TFA and desalted using self-made C18 Stagetips prior to analysis by mass spectrometry.

Crosslinking *E. coli* lysate – *E. coli* lysate was prepared as previously described[1]. Protein concentration was determined by Bradford assay. Crosslinking was carried out on 18.6 mg of protein (0.93 mg/mL), using a crosslinker-to-protein w/w ratio of 0.44:1. UCCL dissolved in 1 mL DMF was diluted with 9 mL crosslinking buffer (50 mM HEPES-OH, 100 mM KCl, 5 mM $MgCl_2$, 5% glycerol, 0.5 mM EDTA, 1 mM DTT, pH 7.4) and added to 10 mL protein solution. Crosslinking was allowed to proceed for 45 min at room temperature, after which the crosslinking reaction was quenched through addition of 1M ABC (1 mL) and incubation for 15 min at room temperature. Crosslinked proteins were precipitated in ice-cold acetone (4 parts acetone to 1 part crosslinked protein solution) and stored overnight at -20 °C. Precipitated proteins were recovered by centrifugation at 14,000 rpm for 20 min. The supernatant was discarded, and the remaining protein pellet was digested following the SPEED protocol[2] with addition of trypsin and overnight incubation at 37 °C.

Crosslinked peptides were enriched by size-exclusion chromatography using a Superdex Peptide 3.2/300 column (GE Healthcare) with a mobile phase consisting of 30% (v/v) acetonitrile and 0.1% TFA, at a flow rate of 10 µL/min.

LC MS^n analysis

Protein digests were analyzed using an Ultimate 3000 RSLC nano system (Dionex, Thermo Fisher Scientific, Germany) coupled on-line to an Orbitrap Fusion Lumos Tribrid mass spectrometer equipped with an EasySpray source and a UVPD module (Thermo Fisher Scientific, Germany) featuring a 213 nm solid-state Nd:YAG laser head (CryLaS GmbH). Peptides were resuspended in 3.2% acetonitrile, 0.1% formic acid, loaded onto and eluted from a 500 mm C18 LC column (75 µm i.d., 2 µm particles, 100 Å pore size) operating at 45 °C, at 300 nL/min, directly into the mass spectrometer. Mobile phase A was 0.1% formic acid and mobile phase B was 80% acetonitrile/0.1% formic acid.

Peptides were analyzed using a “high-high” approach for all acquisition strategies (unless stated otherwise), with both MS1 and MS2 scans carried out in the Orbitrap. MS1 spectra were acquired

at 120,000 resolution, in top-speed data-dependent mode, with monoisotopic peak determination set to “peptide”.

HSA HCD-MS2 acquisition. Crosslinked HSA peptide digest was eluted from the LC column into the mass spectrometer following a linear gradient of 2-40% B over 109 min. Survey spectra were acquired using the following settings: Scan range 400-1600, RF lens 40%, precursor automatic gain control (AGC) target set to 2 x 10^5 and maximum injection time of 50 ms). Precursor isolation on precursor charge states between 3-6 (precursor priority given to the highest charge state) was achieved using the quadrupole, an isolation window of 1.6 *m/z*, an AGC target of 5 x 10^4 and a maximum injection time of 70 ms. HCD was used for peptide fragmentation, with normalized collision energy (NCE) set to 30%. Dynamic exclusion was set to 30 s.

HSA dual MS2, HCD-UVPD-MS2 acquisition. Peptide digest was eluted from the LC column into the mass spectrometer using a nonlinear gradient: 2-4% B in 1 min, 4-6% B in 2 min, 6-37.5% B over 107 min, 37.5-42.5% B in 10 min, 42.5-47.5% B in 5 min and finally 47.5-90% B in 7.5 min. Survey spectra were acquired using the following settings: Scan range 300-1500, RF lens 40%, mass range “normal”, Normalized AGC target 200% (AGC target, 8 x 10^5) and maximum injection time 54 ms. Precursor isolation on precursor charge states 3-8 was achieved using the quadrupole, an isolation window of 1.0 *m/z*, an intensity threshold of 5 x 10^4 (normalized AGC target set to 100%) and dynamic exclusion 60 s. The MS2 AGC target was set to “Standard” using the “Auto” maximum injection time mode. We used a dual MS2 acquisition approach, whereby sequential HCD-MS2 and UVPD-MS2 scans were acquired for each precursor. Normalized collision energy was set to 30% for HCD-MS2 scans with varying 213 nm laser excitation times (1, 5, 10, 20, 30, 40, 50, 100 and 200 ms) for the different UVPD-MS2 experiments. A dual MS2, HCD-HCD-MS2 acquisition strategy was applied to provide non-UVPD reference data. Duplicate runs for each dual MS2 acquisition strategy were performed. MS2 scans were acquired at a resolution of 30,000.

***E.coli* lysate acquisition strategies.** SEC fractions from crosslinked *E.coli* lysate peptide digests were sprayed directly into the mass spectrometer using gradient elution from the LC column, with gradients optimized for individual fractions (from 2-18% mobile phase B to 37.5-46.5% over 90 min, followed by a linear increase to 45-55 and 95% over 2.5 min each). Survey spectra were acquired using the following settings: Scan range 400-1600 *m/z*, Maximum injection time 50 ms, RF lens 35%, Minimum intensity 2.5 x 10^4 and Exclusion duration 60 s. Data decision tree logic was applied for MS2 scan triggering[3,4], with fragmentation by stepped HCD using collision energies of 26, 28 and 30%. Precursor priority was given to the most intense ions. Ion isolation

was carried out in the quadrupole with an isolation window of 1.4 *m/z*. MS2 scans (except where stated otherwise) were analyzed in the Orbitrap at 60,000 resolution, including charge states 3-7, scan range of 150-2000 *m/z* and maximum injection time set to 118 ms. Normalized AGC target was set to 200% (1 x 10^5).

Samples were analyzed following two acquisition strategies, (1) HCD-MS2 and (2) HCD-UVPD-MS2-HCD-MS3 (MS3 scans targeting UVPD-MS2 cleaved UCCL signature peaks). In the HCD-MS2 approach, data were first generated with the purpose of creating a protein database for analyzing subsequent acquisition strategies. For this, survey spectra were acquired using a normalized AGC target of 200% (8 x 10^5), including charge states 2-7. MS2 scans were then analyzed in the ion trap (scan rate "normal", maximum injection time 35 ms). Next, crosslinked peptide search-specific data was generated, acquiring survey scans with a normalized AGC target of 250% (1 x 10^6), including charge states 3-7. The UVPD activation time was 20 ms for the UVPD-MS2 scans. In the HCPD-UVPD-MS2-HCD-MS3 approach, the targeted mass differences for triggering HCD-MS3 scans were ΔM 103.0548, 104.0626, 105.0704 and 106.0783 Da. Dynamic exclusion was set to 2 s and mass tolerance was 8 ppm. MS3 scans were performed on the most intense ion in the pair on the condition that both ions must be of the same charge state. MS3 scans were analyzed in the ion trap using the rapid scan rate, an isolation window of 2 *m/z*, scan range of 150-2000, with normalized AGC target set to 100% and maximum injection time set to 35 ms. Fragmentation was achieved using HCD collision energy at 30%.

Data Analysis

We reanalyzed the publicly available crosslinking data from *E. coli* (DSSO, PXD019120), *D. melanogaster* extract (DSBU, PXD012546) and Synaptosome (DSSO, PXD010317 & PXD015160) datasets. The DSSO and DSBU datasets were analyzed as previously described[5]. Mass spectrometry raw data were preprocessed using a custom python script (https://github.com/Rappsilber-Laboratory/preprocessing). Preprocessing included conversion to MGF file format using MSconvert[6] with subsequent *m/z* recalibration of both MS2 precursor and fragment peaks by employing a linear peptide search to determine the median mass error. The spectra from acquisitions containing multiple MS2 or MS3 were split into separate MGF files for each fragmentation method and MS level. HCD spectra from the HSA-UCCL dataset were additionally denoised to the top 20 peaks per 100 *m/z.* The recalibrated HCD-MS2 spectra were then searched using xiSEARCH[7] 1.7.6.1 using the following parameters: MS1 error tolerances of 3 ppm; MS2 error tolerance of 10 ppm for the HSA-UCCL data and 5 ppm for the *E. coli*-UCCL

data; up to two missing precursor isotope peaks; tryptic digestion specificity with up to four missed cleavages for HSA and two for *E. coli* data; ion series: peptide, b- and y-type; crosslinker: UCCL (mass modification: 250.012224 Da); modifications: carbamidomethylation (Cys, +57.021464 Da) as fixed and oxidation (Met, +15.994915 Da), deamidation (only in *E. coli*; Asn and Gln, +0.984016 Da), amidated and hydrolyzed crosslinker (Lys and protein N-terminus, UCCL-NH2: +267.038773 Da, UCCL-OH +268.022789 Da) as variable modifications; losses: –CH3SOH, –H2O, and –NH3. Crosslink sites for both reagents were allowed for side chains of Lys, Tyr, Ser, Thr, and the protein N-termini. For the *E. coli* UCCL dataset, we included a "non-covalent crosslinker" with zero mass to identify spectra potentially arising from noncovalently associated peptides[8]. MGF files from HSA-UCCL datasets were searched against the sequence of HSA (UniProt P02768). The protein database used for the *E.coli* lysate crosslinked peptide search was created by first searching MS data including charge states 2-7 against the full *E.coli* K12 proteome (UniProt curated reference proteome UP000000625, retrieved on 10/02/2020) using MaxQuant (version 1.6.12.0) with the following settings: Trypsin digestion with maximum two missed cleavages, a maximum of five modifications per peptide (variable modifications: oxidation on methionine, acetylated protein N-termini, UCCL-NH2 (+267.038773) and UCCL-OH (+268.022789); fixed modifications: cysteine carbamidomethylation), MS1 match tolerance 20 ppm (first search)/4.5 ppm (main search), MS/MS match tolerance 0.5 Da and 1% FDR on PSM protein group level. This resulted in the identification of 2314 proteins, which subsequently formed the protein database used for crosslinked peptide search.

The HSA-UCCL search results were split by excitation time, and a 5% CSM level FDR was applied to each excitation time set. The "sequence-consecutive peptides" and "minimum peptide length of 5 amino acids" filters were applied. To further minimize false positives post-FDR, resulting crosslinks were mapped onto the crystal structure (PDB ID: 1AO6) and all CSMs corresponding to overlength (>30 Å) and unknown distances crosslinks were discarded. CSMs for which no UVPD spectrum had been acquired were excluded from further analysis. The UVPD spectra corresponding to the remaining CSMs were then annotated with pyXiAnnotator (https://github.com/Rappsilber-Laboratory/pyXiAnnotator/) v0.3.5 with the cleavable crosslinker stub fragments A: 41.002739665 Da, B: 72.974810365 Da, C: 177.037410645 Da, and D: 209.009481345 Da, as well as their hydrogen-shifted variants: -2, -1, +1, and +2 using a fragment tolerance of 15 ppm.

The UCCL *E. coli* lysate search results were filtered prior to FDR to matches having a minimum of three matched fragments per peptide, and a delta score of >15% of the match score. A 5%

CSM level FDR with sequence-consecutive and minimum peptide length (5 amino acids) filters, was applied. CSMs for which no UVPD spectrum had been acquired were excluded. The precursor *m/z* window (-1 to +1.5 *m/z*) was cut out of the UVPD spectra prior to further analyses. The UVPD spectra were then annotated using pyXiAnnotator according to the identification of the corresponding HCD CSMs with a-, b-, and y-ion series and the cleavable crosslinker stub fragments A, B, C, and D including the hydrogen-shifted variants of the B and C stubs using a 10 ppm fragment mass tolerance.

The geometric medians of the stub fragment to backbone fragment distributions were calculated using the “geom_median” python package[9]. For the doublet rank evaluation, the “deisotoped max rank” column from pyXiAnnotator output was used which determines the rank of the annotated isotope cluster by comparing the maximum intensity peak of each isotope cluster. Doublet rank was then assigned by the higher of the two doublet peak ranks. To evaluate if the correct peaks were triggered for MS3, the MS3 precursor *m/z* was extracted from the scan header of MS3 spectra associated with the unique CSMs passing FDR (as described above) and compared with the corresponding UVPD annotation result. If the MS3 precursor matched a crosslinked peptide stub fragment within 20 ppm error tolerance, it was assigned as correctly triggered. For the evaluation of the MS3 trigger specificity, the number of MS3 scans associated with non-unique CSMs and linear PSMs (with and without hydrolyzed or amidated crosslinker modifications) passing the FDR threshold was used.

Settings for xiDOUBLET doublet detection algorithm used for the UCCL dataset were: ms2_tol of 5 ppm tolerance; crosslinker UCCL; stubs B & C with -1 and +1 hydrogen_shift; rank_cutoff of 20; cap of 4; second_peptide_mass_filter 500; mz_window_filter 1.5. The DSSO dataset was analyzed with comparable xiDOUBLET settings as previously described[10].

The mass spectrometry proteomics data have been deposited to the ProteomeXchange Consortium via the PRIDE[11] partner repository with the dataset identifier PXD040267 and 10.6019/PXD040267.

Figures

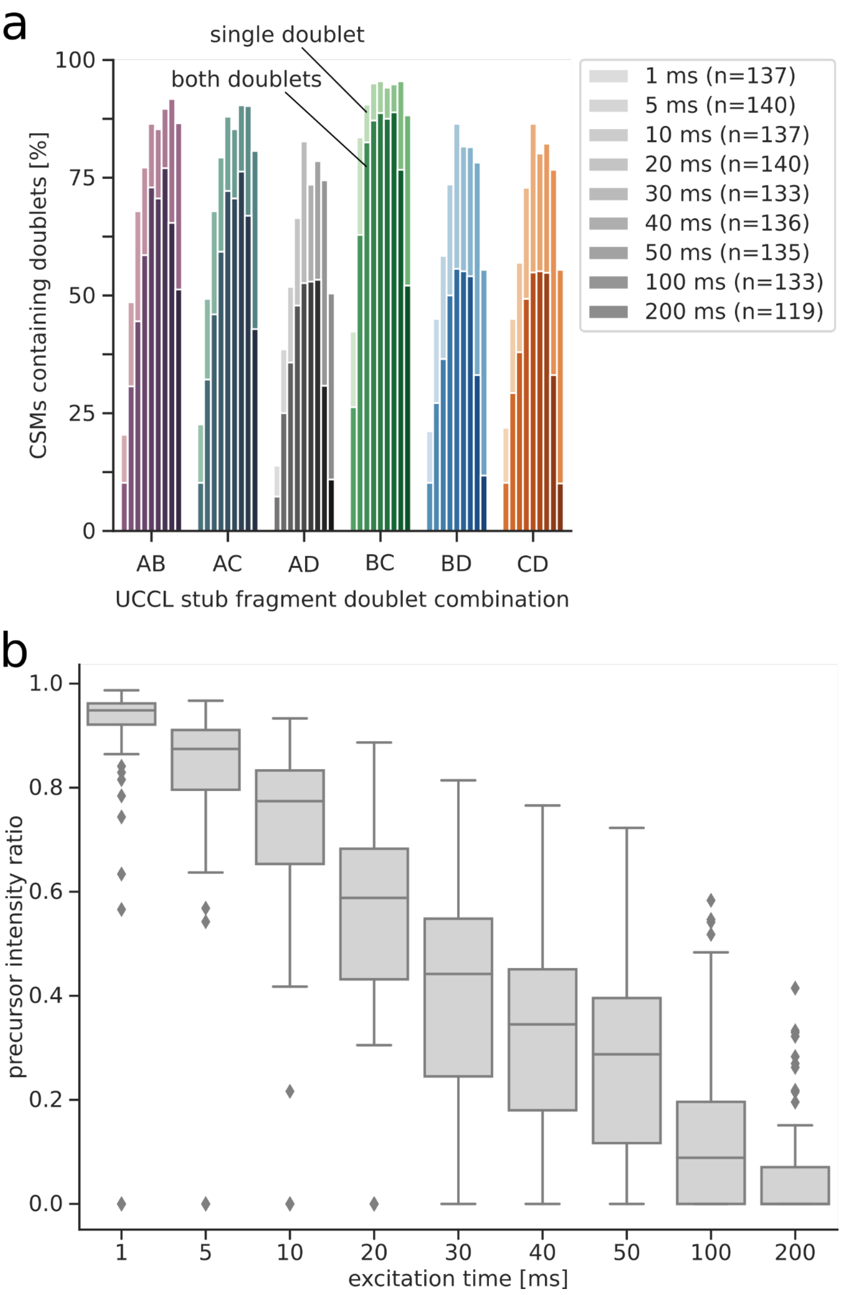

Figure S1. UVPD cleavage. (a) Proportion of HSA CSMs containing doublets of all possible combinations of the four UCCL peptide stub fragments over varying excitation times (1 - 200 ms). Proportions for CSMs containing only a single peptide doublet for one of the crosslinked peptides are plotted in lighter shades on top of the proportions of CSMs containing both doublets for both crosslinked peptides. (b) Precursor intensity ratio (unfragmented precursor intensity in MS2 divided by total MS2 intensity) over varying excitation times, showing the cleavage efficiency of UVPD. Boxplot depicts the median (middle line), upper and lower quartiles (boxes), and 1.5 times the interquartile range (whiskers).

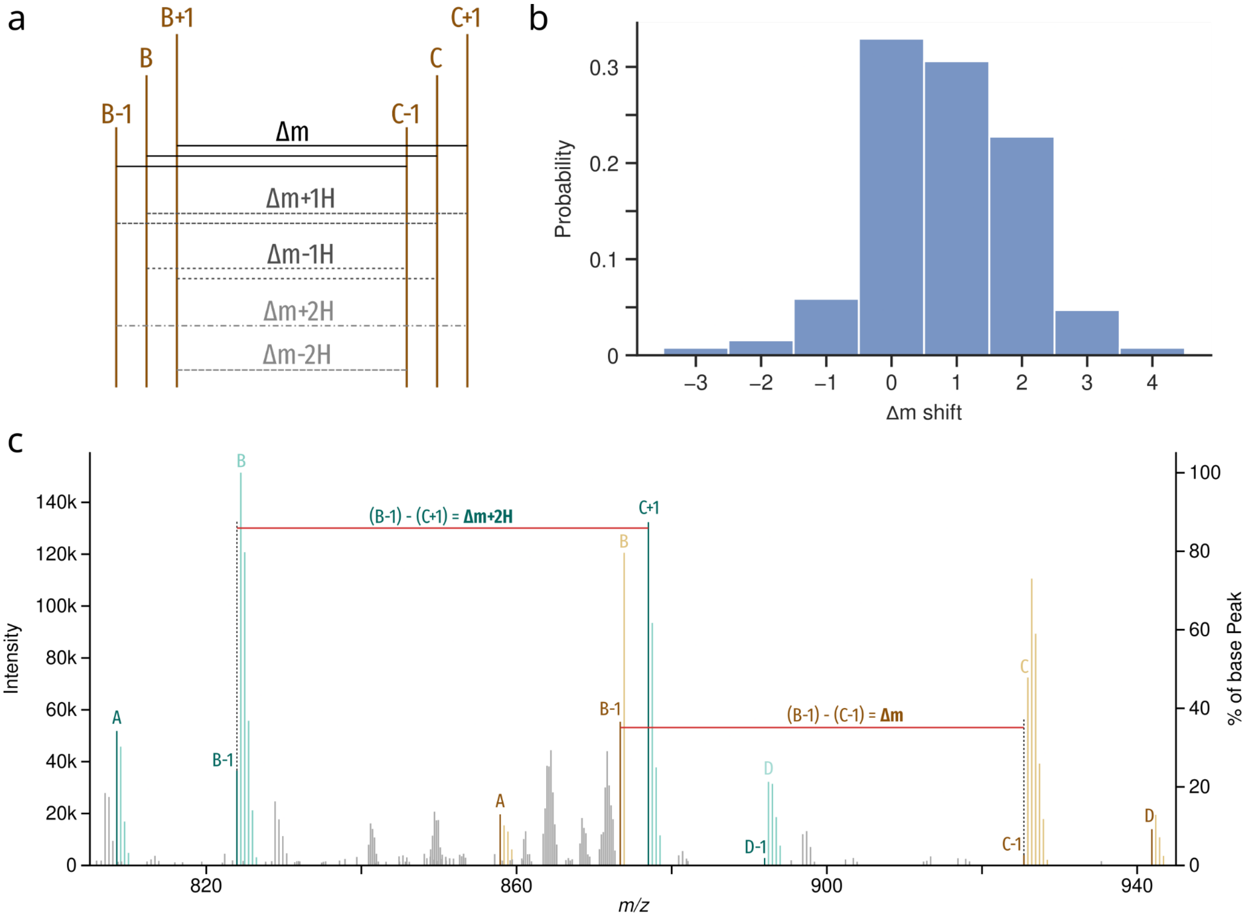

Figure S2. Hydrogen shift variants of cleavable crosslinker peptide stub fragments. (a) Schematic representation of possible delta masses between different hydrogen enriched or depleted variants of peptide stub fragments. (b) Histogram of observed delta mass shifts of the most intense doublet for each peptide from the 20ms UVPD excitation time HSA-UCCL data. (c) Example spectrum showing doublets with different delta masses due to hydrogen shift peaks.

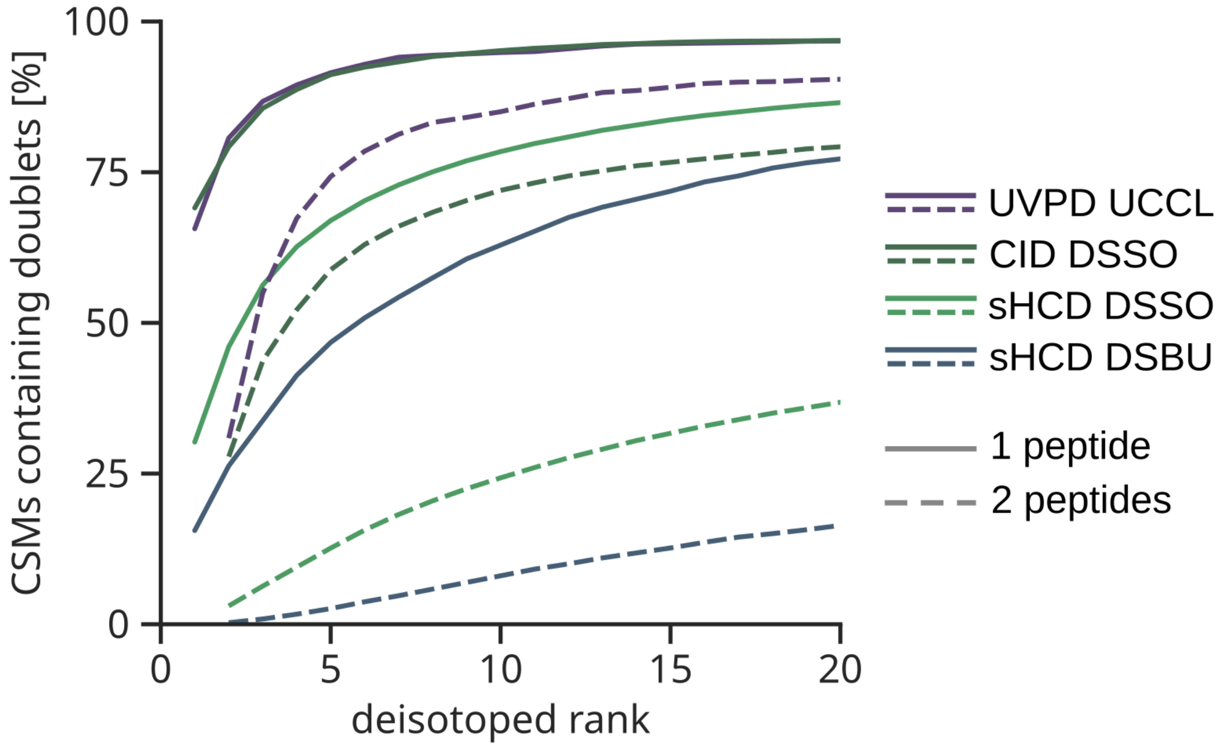

Figure S3. Proportion of CSMs containing doublets passing different intensity rank cut-off for all four analyzed datasets.

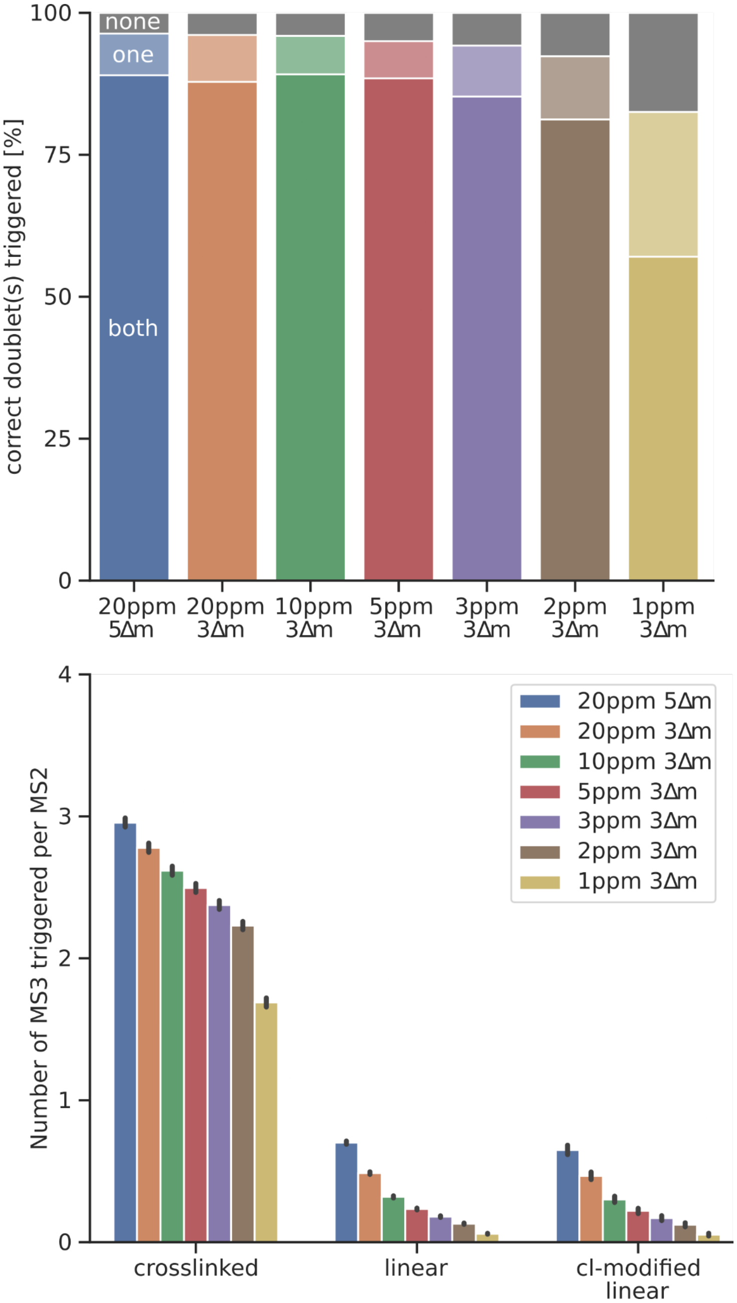

Figure S4. Influence of the number of delta masses (3 or 5) and mass tolerance (1 - 20 ppm) on doublet detection sensitivity and specificity for the xiDOUBLET algorithm.

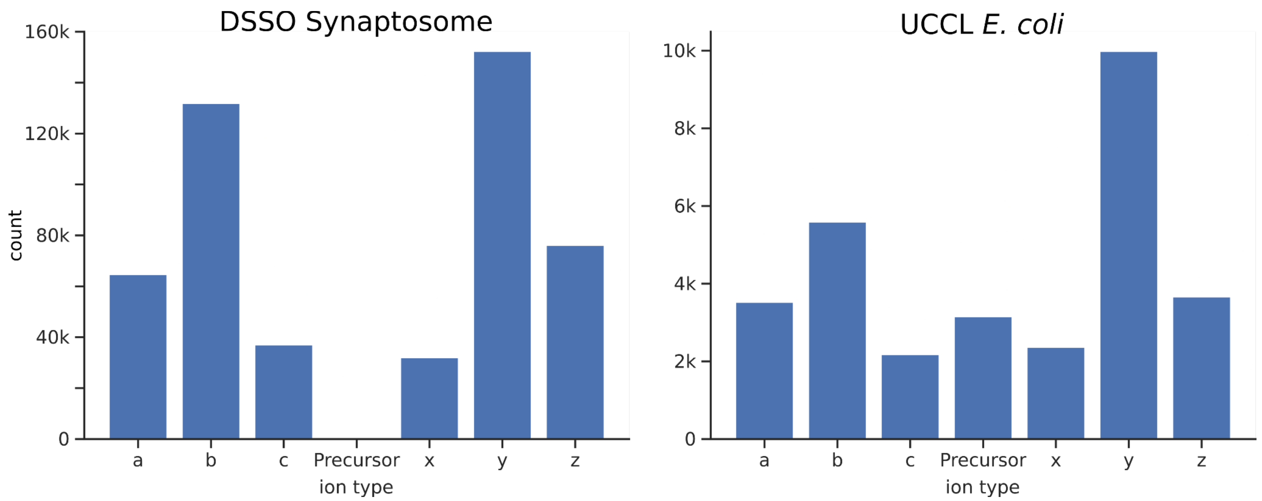

Figure S5. Observed ion types in the MS3 spectra of identified crosslinked peptides for the DSSO synaptosome dataset (CID MS2 -> CID MS3) and UCCL *E. coli* dataset (UVPD MS2 -> HCD MS3).

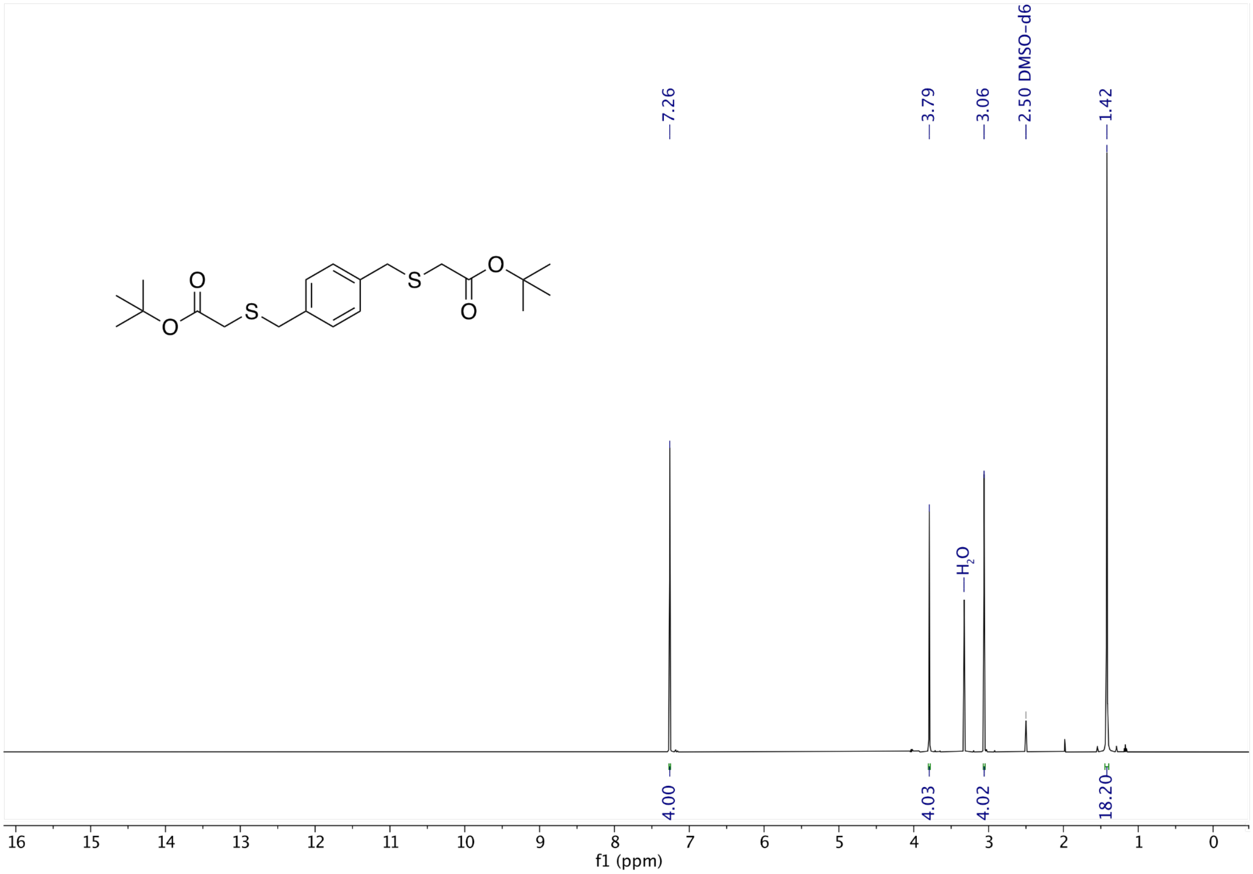

Figure S6. ^{1}H NMR spectrum of **1**.

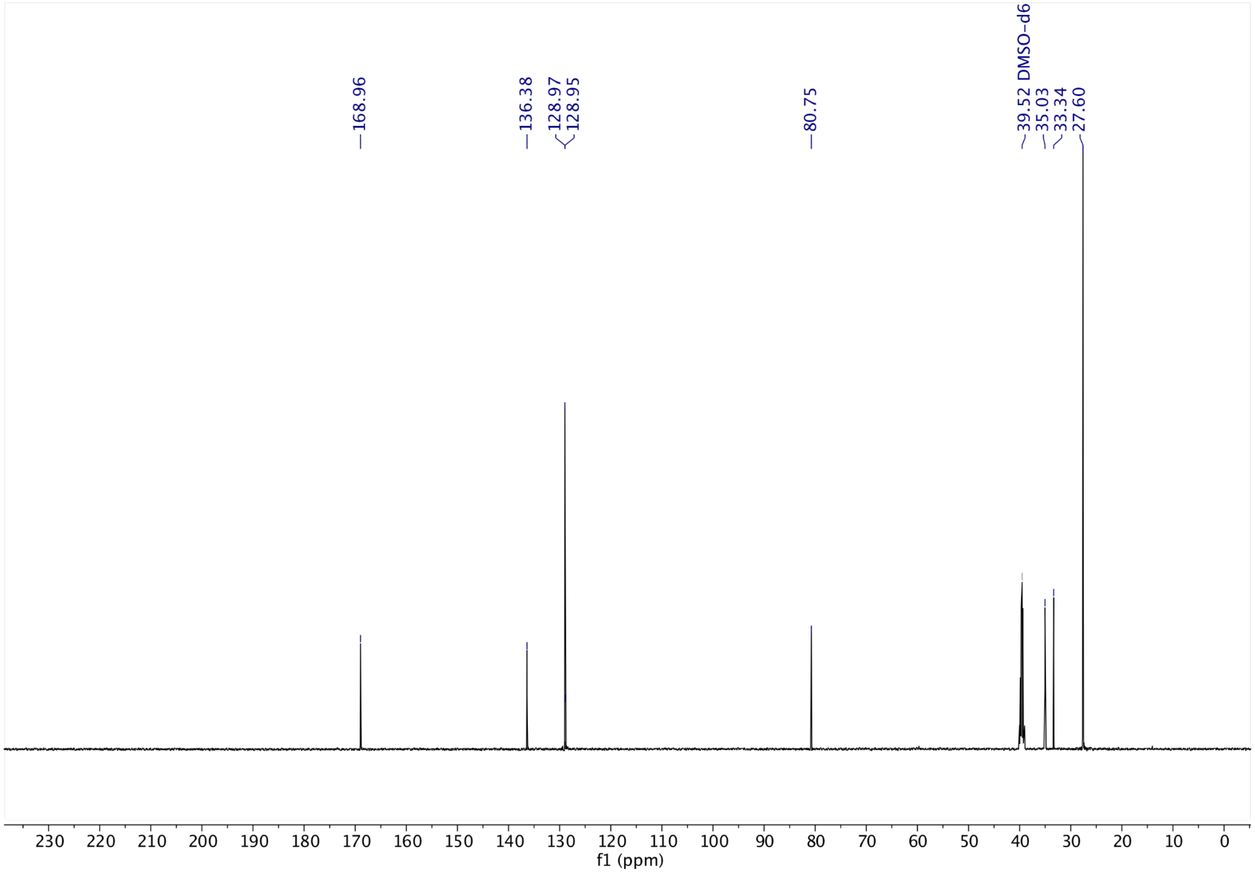

Figure S7. ^{13}C NMR spectrum of **1**.

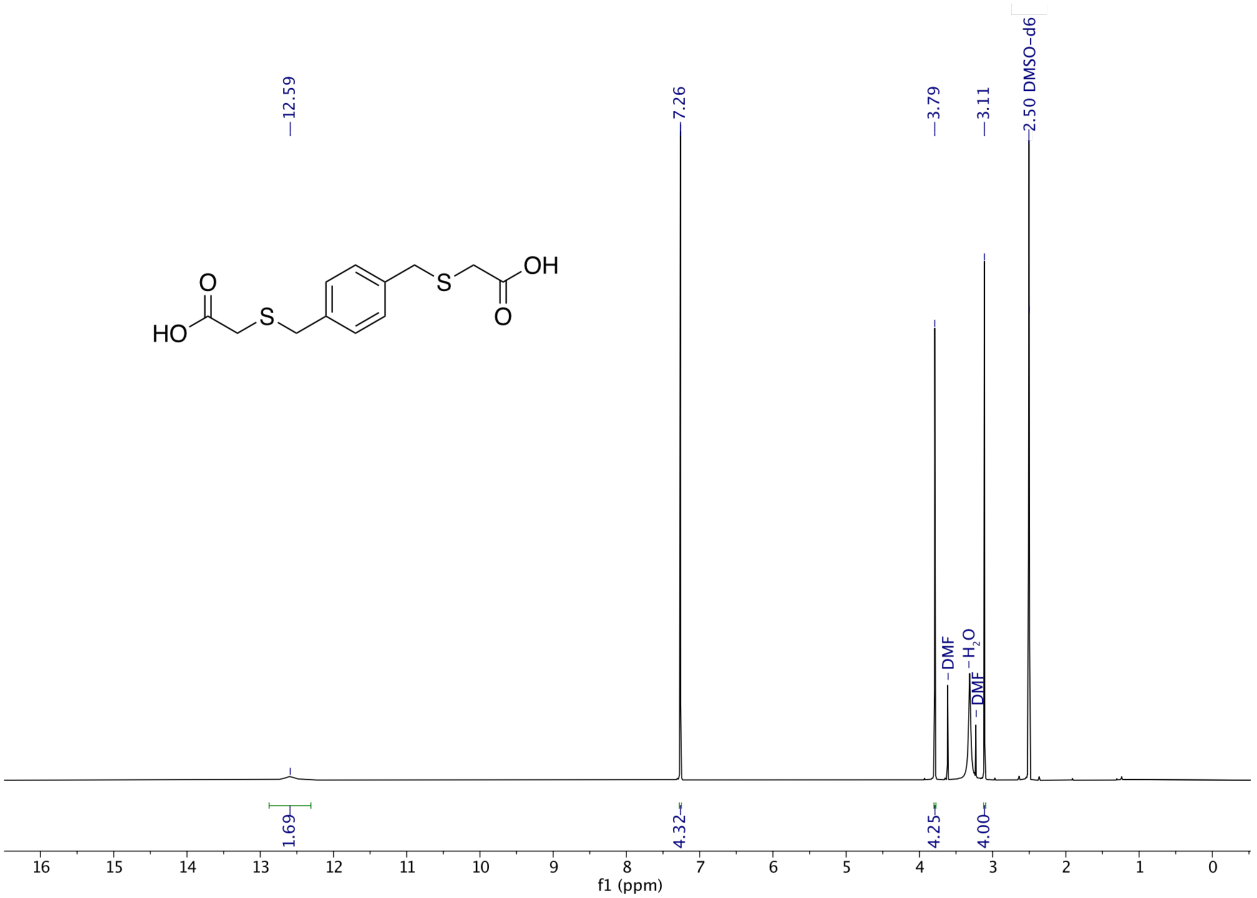

Figure S8. ^{1}H NMR spectrum of **2**.

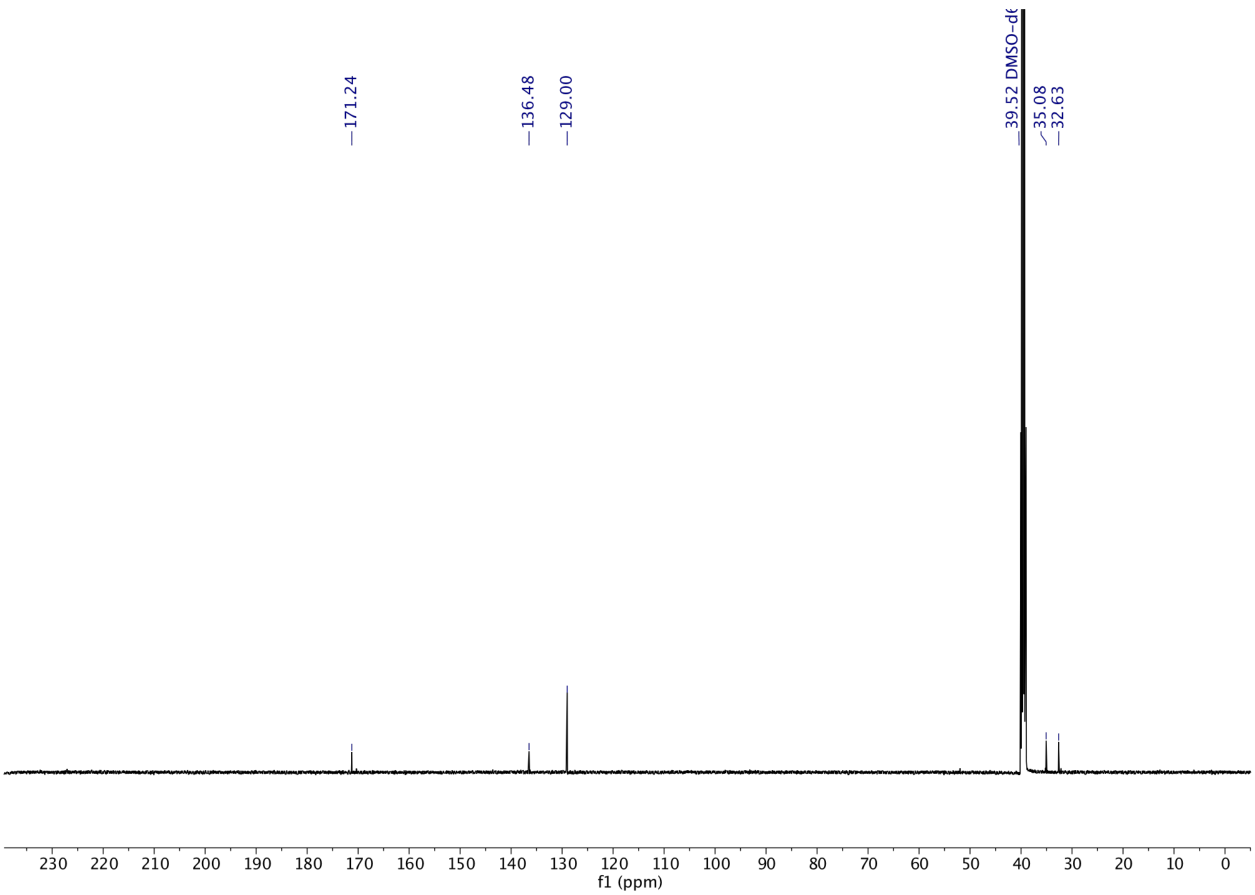

Figure S9. ^{13}C NMR spectrum of **2**.

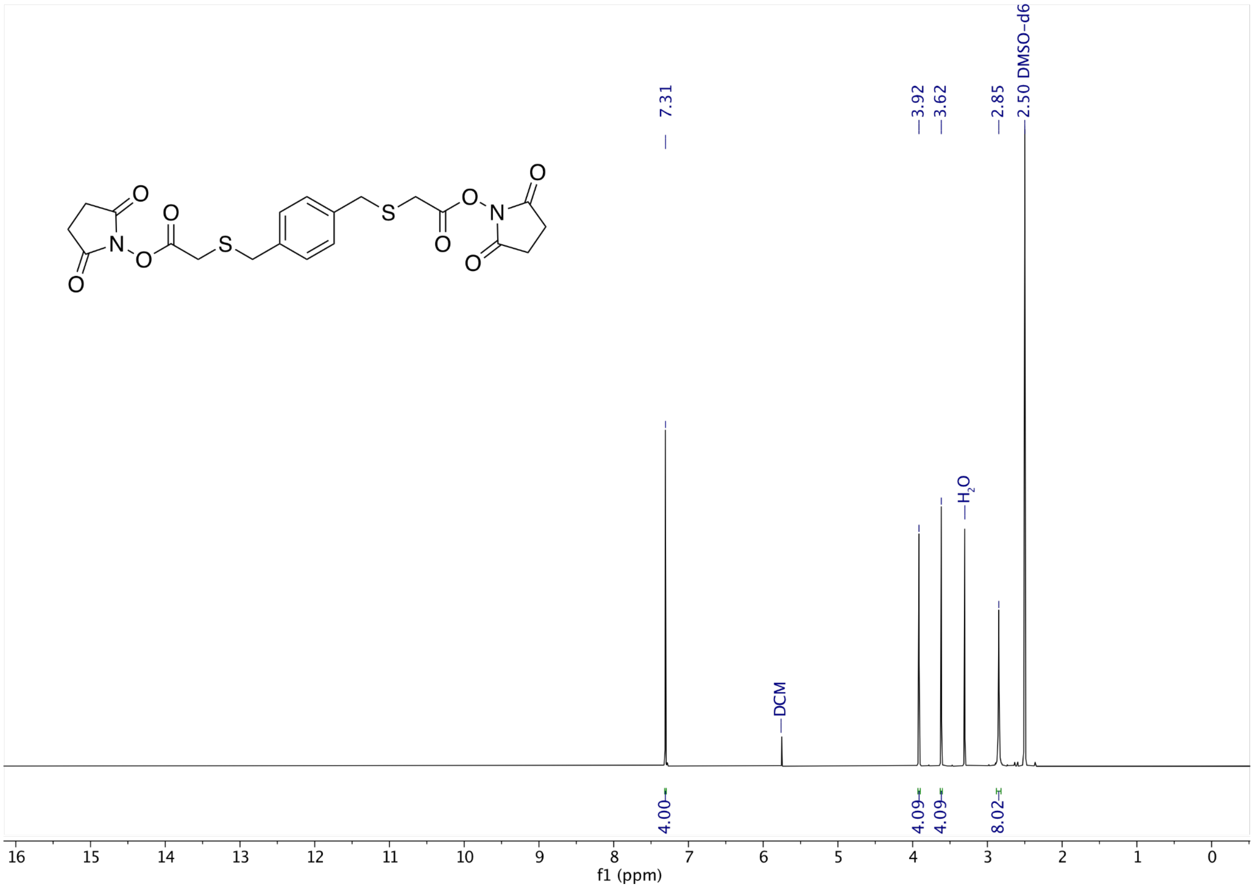

Figure S10. ^{1}H NMR spectrum of UCCL.

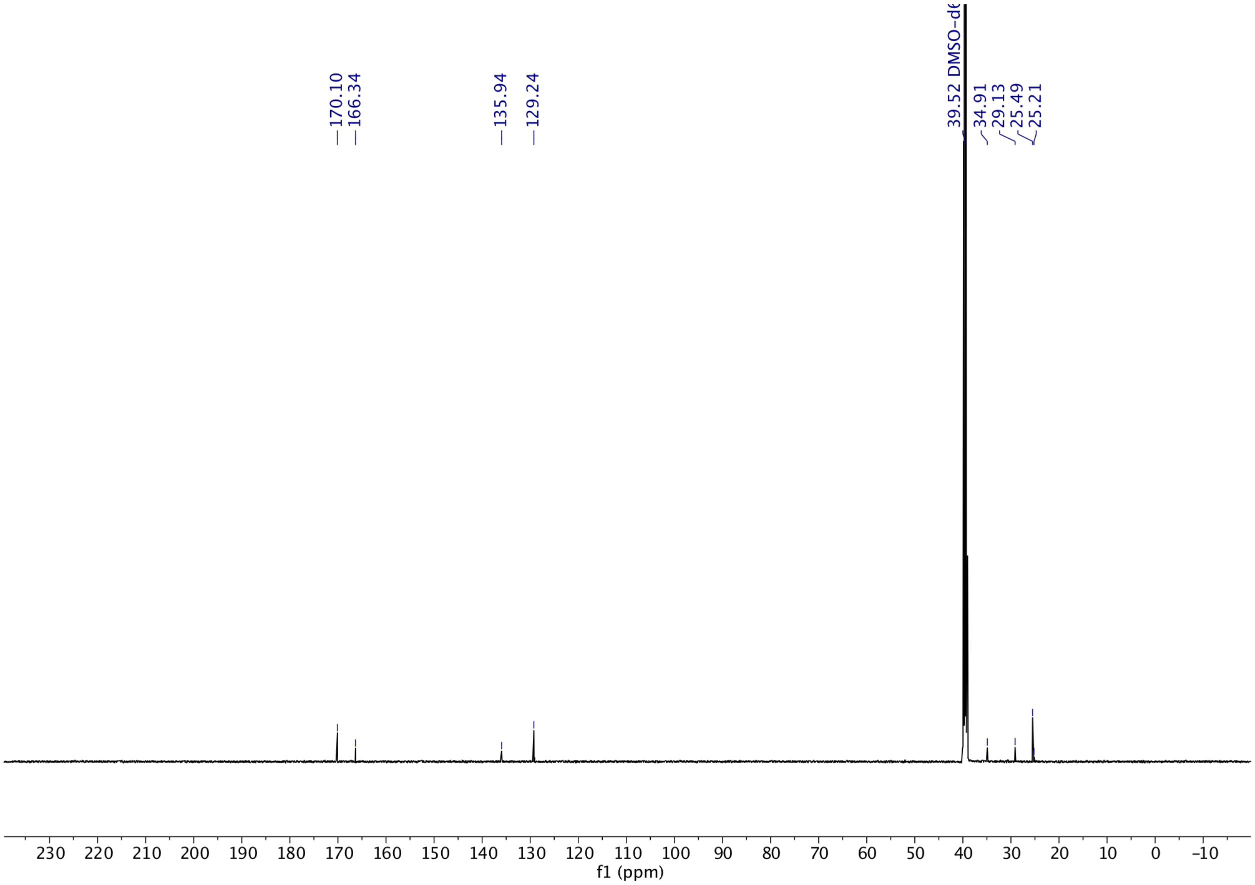

Figure S11. ^{13}C NMR spectrum of UCCL.

Supplemental References

1. Lenz, S. *et al.* Reliable identification of protein-protein interactions by crosslinking mass spectrometry. *Nat. Commun.* **12**, 3564 (2021).
2. Doellinger, J., Schneider, A., Hoeller, M. & Lasch, P. Sample Preparation by Easy Extraction and Digestion (SPEED) - A Universal, Rapid, and Detergent-free Protocol for Proteomics Based on Acid Extraction. *Mol. Cell. Proteomics* **19**, 209–222 (2020).
3. Giese, S. H., Belsom, A. & Rappsilber, J. Optimized Fragmentation Regime for Diazirine Photo-Cross-Linked Peptides. *Anal. Chem.* **88**, 8239–8247 (2016).
4. Kolbowski, L., Mendes, M. L. & Rappsilber, J. Optimizing the Parameters Governing the Fragmentation of Cross-Linked Peptides in a Tribrid Mass Spectrometer. *Anal. Chem.* **89**, 5311–5318 (2017).
5. Kolbowski, L. *et al.* Improved Peptide Backbone Fragmentation Is the Primary Advantage of MS-Cleavable Crosslinkers. *Anal. Chem.* **94**, 7779–7786 (2022).
6. Holman, J. D., Tabb, D. L. & Mallick, P. Employing ProteoWizard to Convert Raw Mass Spectrometry Data. *Curr. Protoc. Bioinformatics* **46**, 13.24.1–9 (2014).
7. Mendes, M. L. *et al.* An integrated workflow for crosslinking mass spectrometry. *Mol. Syst. Biol.* **15**, e8994 (2019).
8. Giese, S. H., Belsom, A., Sinn, L., Fischer, L. & Rappsilber, J. Noncovalently Associated Peptides Observed during Liquid Chromatography-Mass Spectrometry and Their Effect on Cross-Link Analyses. *Anal. Chem.* **91**, 2678–2685 (2019).
9. Pillutla, K., Kakade, S. M. & Harchaoui, Z. Robust Aggregation for Federated Learning. *IEEE Trans. Signal Process.* **70**, 1142–1154 (2022).
10. Kolbowski, L., Fischer, L. & Rappsilber, J. Cleavable crosslinkers redefined by novel MS3-trigger algorithm. *bioRxiv* 2023.01.26.525676 (2023) doi:10.1101/2023.01.26.525676.
11. Perez-Riverol, Y. *et al.* The PRIDE database resources in 2022: a hub for mass spectrometry-based proteomics evidences. *Nucleic Acids Res.* **50**, D543–D552 (2022).
